# Simple and reliable direct patterning method for carbon-free solution-processed metal oxide TFTs

**DOI:** 10.1038/s41598-018-31134-w

**Published:** 2018-08-27

**Authors:** Masashi Miyakawa, Mitsuru Nakata, Hiroshi Tsuji, Yoshihide Fujisaki

**Affiliations:** NHK Science & Technology Research Laboratories, Tokyo, 157-8510 Japan

## Abstract

Metal oxide TFT fabrication based on a solution-processing method is considered a promising alternative to conventional vacuum processing and has a number of advantages such as low cost, large-area fabrication, and process simplicity. A simple and reliable, direct patterning method for obtaining a carbon-free aqueous metal oxide film is presented herein. Patterning, which is achieved by selective photoreaction of water molecules under ultraviolet irradiation and by a safe, environment-friendly chemical etching process using a non-toxic organic acid, is followed by an annealing process at a temperature of 350 °C to obtain carbon-free metal oxide TFTs. In–Ga–Zn oxide (IGZO), TFTs on SiO_2_ dielectrics that were fabricated with a direct patterning method exhibited an average mobility of 4.3 cm^2^/V·s with good uniformity, which is comparable to TFTs formed by conventional photolithography. The TFTs exhibited stable performance with small (within 0.5 V) shifts in switch-on voltage under positive and negative bias stress. Fabrication of flexible IGZO TFTs by direct patterning was also achieved.

## Introduction

Thin-film transistors (TFTs) based on metal oxide semiconductors, such as In–Ga–Zn oxide (IGZO), are highly desirable due to their high mobility, high on/off ratio, and transparency^1–4^. Various applications using metal oxide TFTs have been proposed for a wide variety of electronic devices such as rigid and flexible organic light-emitting diode (OLED) displays^5,6^, wearable electronics^7,8^, non-volatile memories^9^, RF-ID tags^10^, and biosensors^11,12^. Metal oxide TFT fabrication based on a solution processing method is a promising alternative to conventional vacuum processing due to its low cost, large-area fabrication, and process simplicity^2,13–19^. Traditional photolithography is time consuming, involves multiple steps, and uses toxic organic-based solvents and strong acids. There is a need for a simple patterning process for device integration. Direct patterning^20–23^, drop-on-demand inkjet-printing^24–26^, flexographic printing^27^, and gravure printing^28^ have been proposed. While the printing process has certain advantages, such as selective deposition and a mask-less process, strict control of printing conditions^13,26^ are difficult to achieve but necessary to obtain a quality printed film of the right shape, size, uniformity, and thickness^22^. Direct patterning is able to achieve fine, uniform patterning because it is an optical process based on photo-selective reaction. Selective reaction and oxidation of the coated film under UV irradiation via a UV mask alter the film’s solubility for subsequent etching. Previous studies on direct patterning techniques used carbon-based photosensitive additives, such as β-diketonato ligand chelate complex (i.e., benzoylacetone and acetylacetone),^20^ to fabricate metal oxide TFTs. However, these TFTs exhibit substandard performance due to insufficient oxidation of the precursor. In general, after direct patterning of the thin film, the film is oxidized via a thermal annealing process to form metal–oxide–metal (M-O-M) bonds via condensation reaction^29^. The ligands in metal salts and solvents must be completely decomposed at that time to produce a high-quality oxide film. Any impurities that remain, such as organic contents, will suppress metal oxide condensation and degrade electrical performance^29,30^. Therefore, a direct patterning method that limits the amount of organic content in the precursor is critical to reducing carbon impurities in the oxide film for improved performance.

The research literature has reported the successful fabrication of metal oxide films for TFT applications by combined UV irradiation and photo-annealing process^31–35^. Short-wavelength deep UV (DUV) irradiation of the precursor, assisted by oxidizing radicals from photochemical activation, is known to be effective in oxidation of the film and decomposition of the ligands in metal salts^31^. Recently, direct patterning of the precursor without the use of photosensitive additives by applying DUV irradiation was reported^22,36^. However, the precursor was still prepared with carbon-based materials, such as organic solvents, additives, and ligands in metal salts.

In this study, a direct patterning method for fabricating carbon-free metal oxide thin films was proven using aqueous metal oxide precursors that were prepared without any carbon-related species. In the proposed method, the water molecules underwent photochemical reaction directly in the aqueous precursor based on free-radical reactions induced by UV irradiation. A safe etching processes based on a non-toxic dilute organic acid (citric acid) was also developed. The entire process did not require any organic solvents. We successfully fabricated carbon-free, solution-processed oxide TFTs by direct patterning, which are comparable in performance to TFTs fabricated by conventional photolithography.

## Results and Discussion

**Figure 1:**
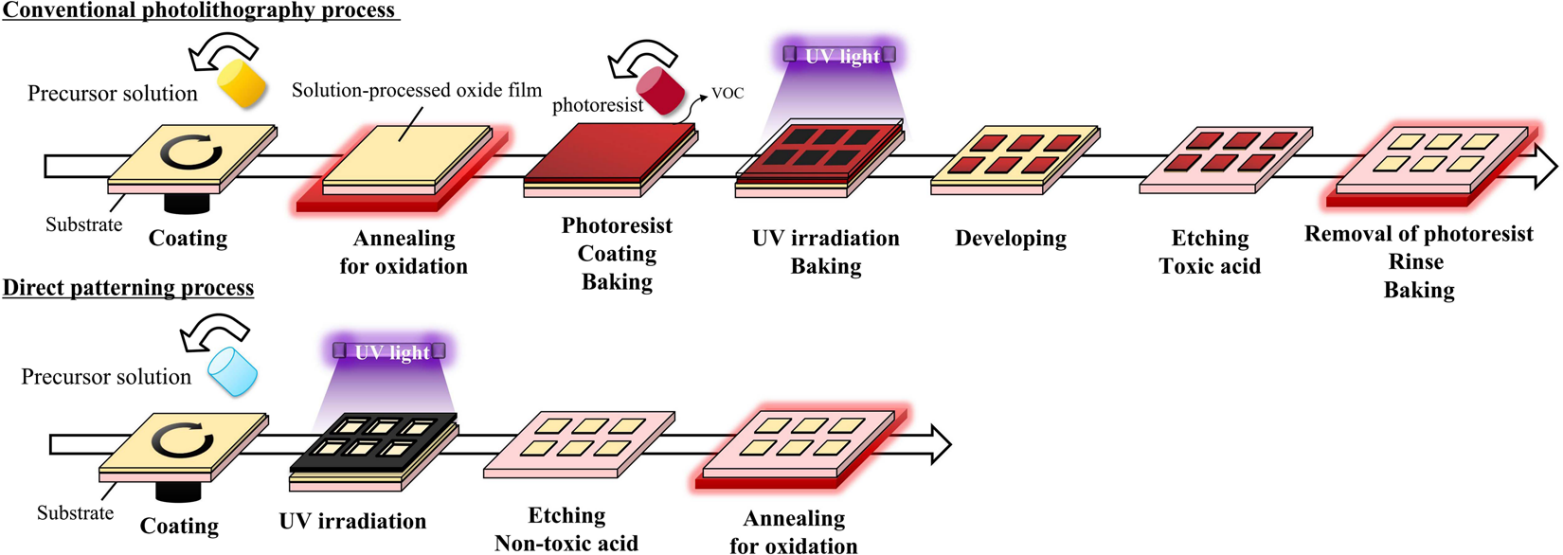
Comparison of process flow for metal oxide film fabrication using a conventional photolithography process vs. direct patterning process.

Figure 1 shows the fabrication process flow for conventional photolithography vs. the proposed direct patterning. Photolithography requires multiple steps (from coating to photoresist removal), involves a time-consuming baking and drying process, and produces a lot of waste, including toxic volatile organic compounds (VOCs) and other waste gases that are emitted into the environment^37^. In contrast, direct patterning is not only much simpler but cuts down on processing time, equipment cost, and chemical usage. Moreover, an aqueous precursor has many environmental benefits, including reductions in VOCs to boot.

**Figure 2:**
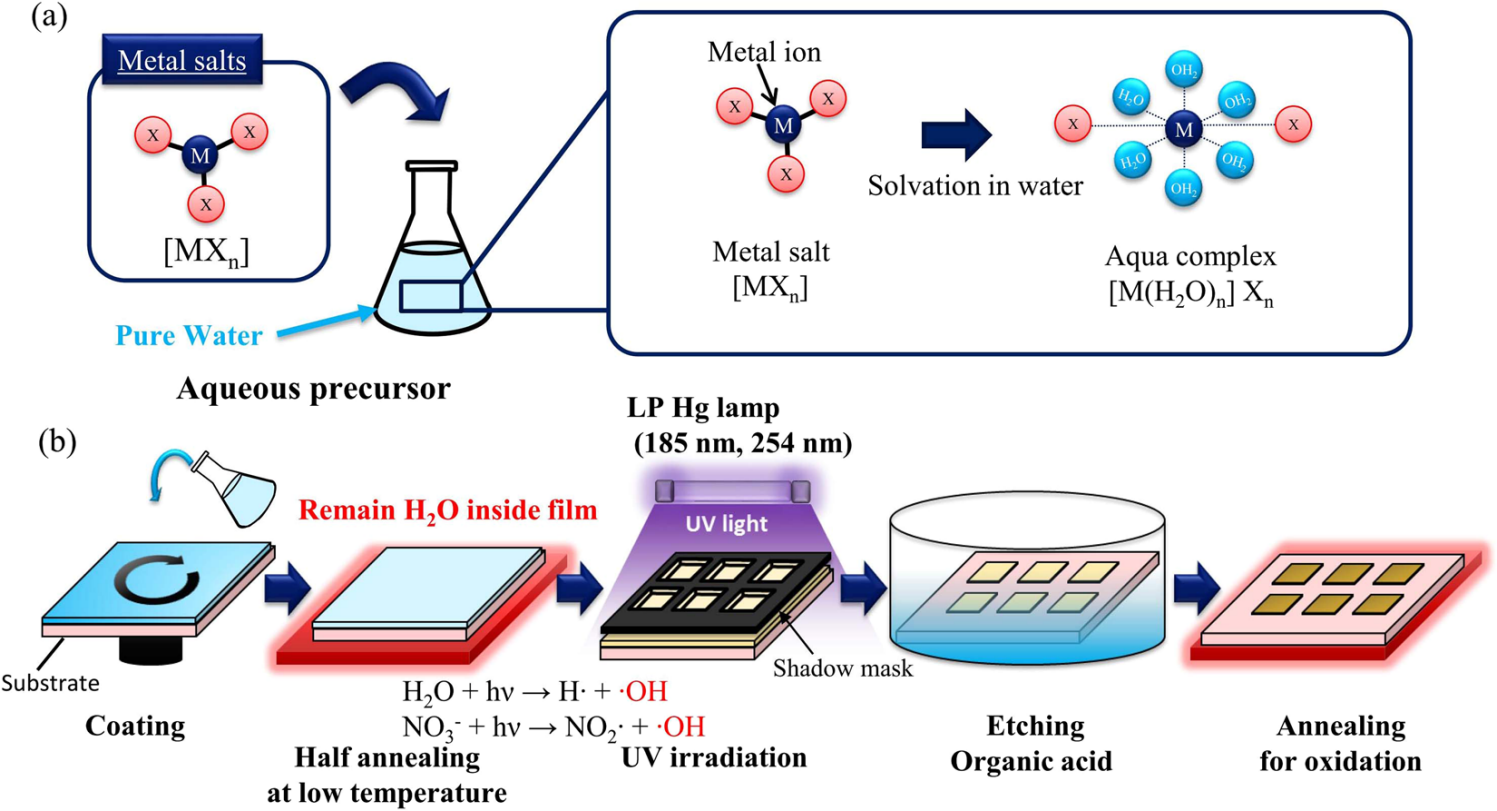
(**a**) Aqua complexes [M(H_2_O)_*m*_]X_*n*_ formed by solvation with water molecules in the aqueous precursor. (**b**) Schematic illustration of the direct photoreactive patterning method.

Figure 2 is a schematic of the proposed direct patterning process based on photochemical reaction of the water molecules. Aqueous precursors have been reported to form aqua complexes such as [M(H_2_O)_*m*_]X_*n*_ during solvation as ligands are replaced by water molecules^30,38^. During photoreactive patterning, the water molecules in the aqua complexes react directly under UV irradiation. To generate photoreaction effectively, a pre-irradiation annealing process ensures that the water molecules remain inside the film in a half-annealed condition. Upon irradiation of these complexes with short-wavelength (185 and 254 nm) UV light, the free-radical reactions of the H_2_O moieties are induced by photodissociation, forming hydroxyl radical species^39,40^. Along with photoreaction of H_2_O, the photo-reaction of nitrate ligands of NO_3_ are initiated as well^33^. The photochemical reaction is written as follows:

$${{\rm{H}}}_{{\rm{2}}}{\rm{O}}+{\rm{hv}}\to {\rm{H}}\cdot +\cdot {\rm{OH}}$$

$${{{\rm{NO}}}_{{\rm{3}}}}^{-}+{\rm{hv}}\to {{\rm{NO}}}_{{\rm{2}}}\cdot +\cdot {\rm{OH}}$$

The generated hydroxyl radical species are known to be strong oxidizing agents for accelerating oxidation reactions^32^. The generated hydroxy radicals assist the M-OH activation, which leads to efficient M-O-M condensation^33^. The condensation reaction for M-O-M bonds is written as follows:

$${\rm{M}} \mbox{-} {\rm{OH}}+{\rm{M}} \mbox{-} {\rm{OH}}\to {\rm{M}} \mbox{-} {\rm{O}} \mbox{-} {\rm{M}}+{{\rm{H}}}_{{\rm{2}}}{\rm{O}}$$

In the proposed patterning method, these photoreactions are utilized directly for patterning. Therefore, we successfully achieved direct patterning of a simple aqueous precursor that did not contain carbon-based photosensitive additives. Selective exposure to UV light was achieved by placing a metal mask on the film during irradiation. Oxidation of the irradiated film altered its solubility, while regions not exposed to UV light were easily solved. Because the aqueous precursor was synthesized from only metal-nitrate salts and pure water, the resulting oxide film was expected to be free of carbon impurities, excepting accidental contamination during fabrication. Moreover, the easy decomposition of the nitrate-based precursor in aqueous systems leads to fewer impurities and efficient M-O-M bonds^35^, resulting in a dense oxide film.

**Figure 3:**
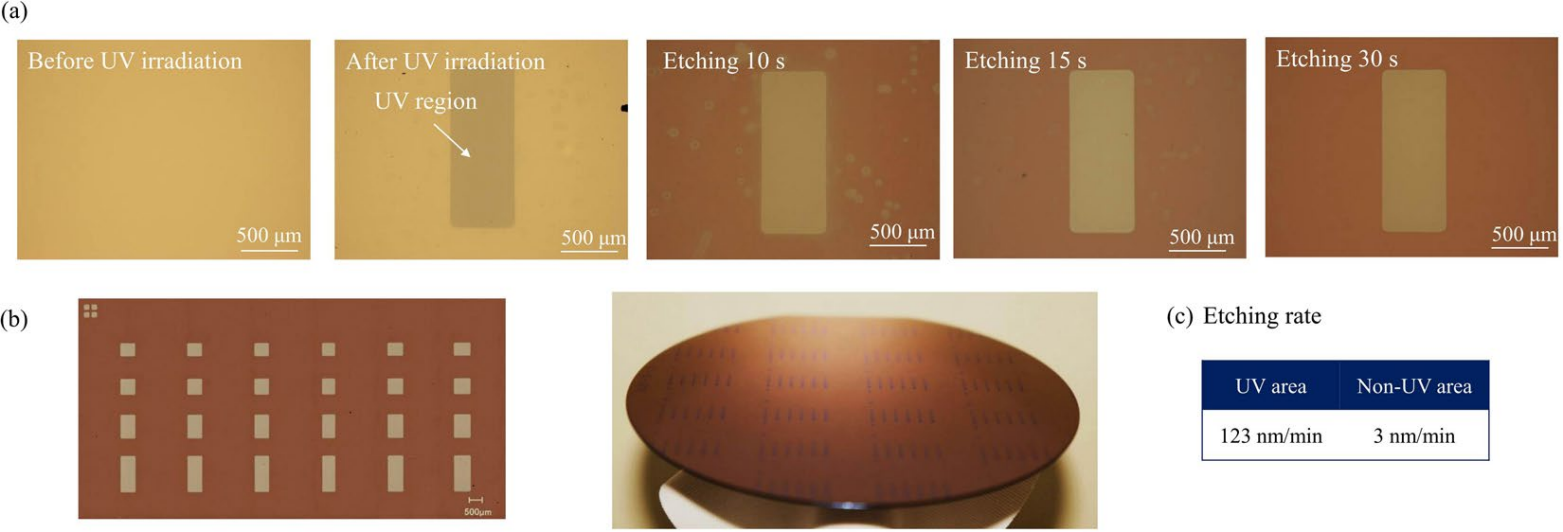
(**a**) Optical microscopy images acquired at different times during the direct phot oreactive patterning of island-shaped IGZO films. (**b**) Photograph of differently sized patterns and IGZO film on a 4-in Si wafer. (**c**) Etching rates of the IGZO films in UV-irradiated and non-irradiated areas.

Figure 3 shows optical microscopy images of the IGZO films fabricated using direct patterning at different etching times. A stable patterning was produced while keeping the shape of the IGZO film intact. We observed high solubility during etching (within about 10 s) in regions not exposed to UV irradiation and high stability in UV-exposed regions. Thus, a uniform and stable patterning was achieved as a result of the difference in etching rate (by a factor of approximately 40) due to differences in the film’s solubility and the photoreactive process to which the aqueous precursor was subjected.

**Figure 4:**
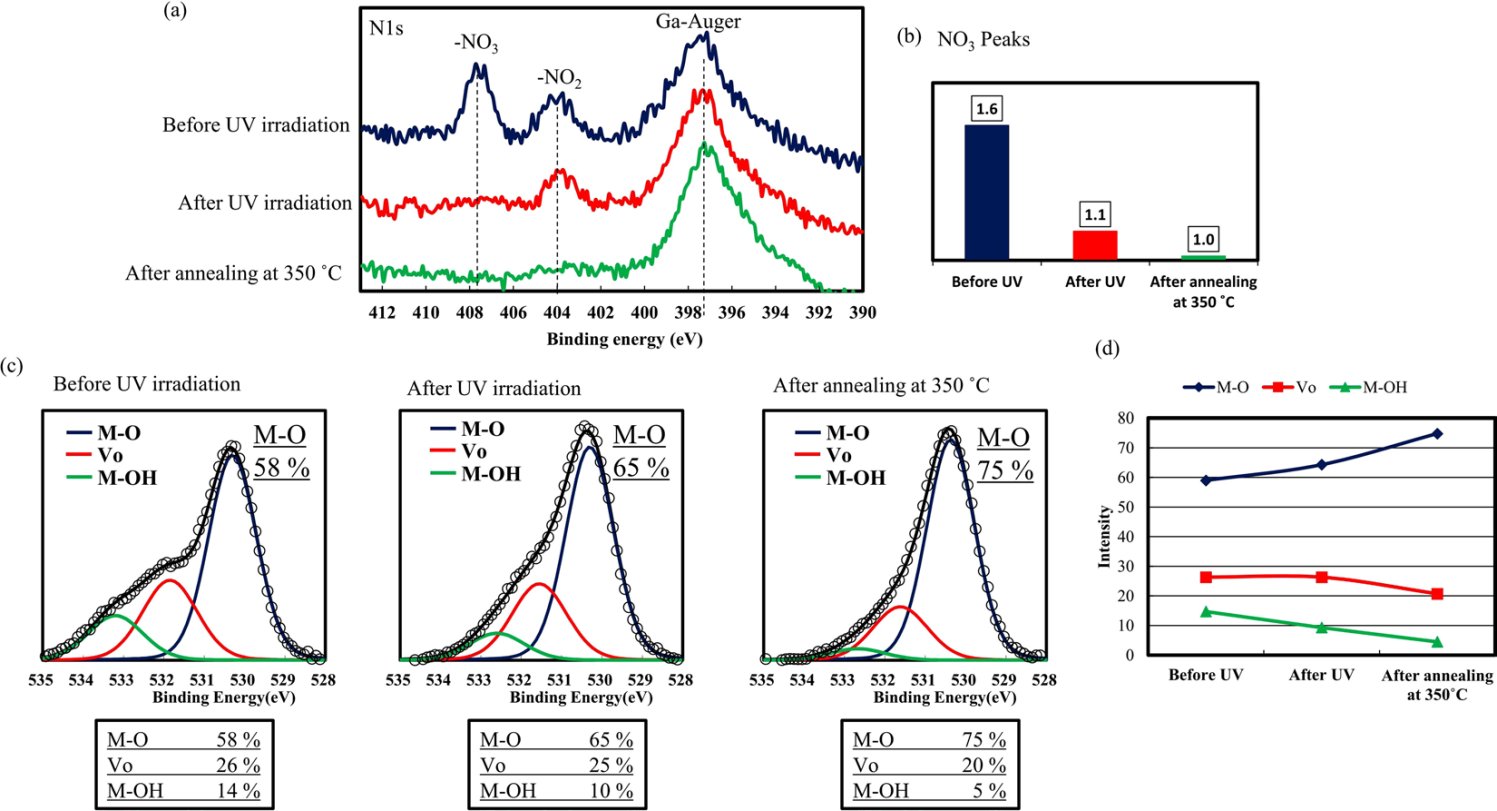
(**a**) XPS N1s spectra of the IGZO films fabricated by the direct-patterning process at the indicated conditions. (**b**) Intensity of NO_3_ peaks in the corresponding IGZO films. (**c**) XPS O1s spectra of the IGZO films at the indicated conditions. The O1s peaks were analyzed by curve-fitting the M-O (lattice oxygen), Vo (oxygen vacancy), and M-OH (hydroxyl) peaks. (**d**) Intensity of the O1s peaks for M-O, Vo, and M-OH.

**Figure 5:**
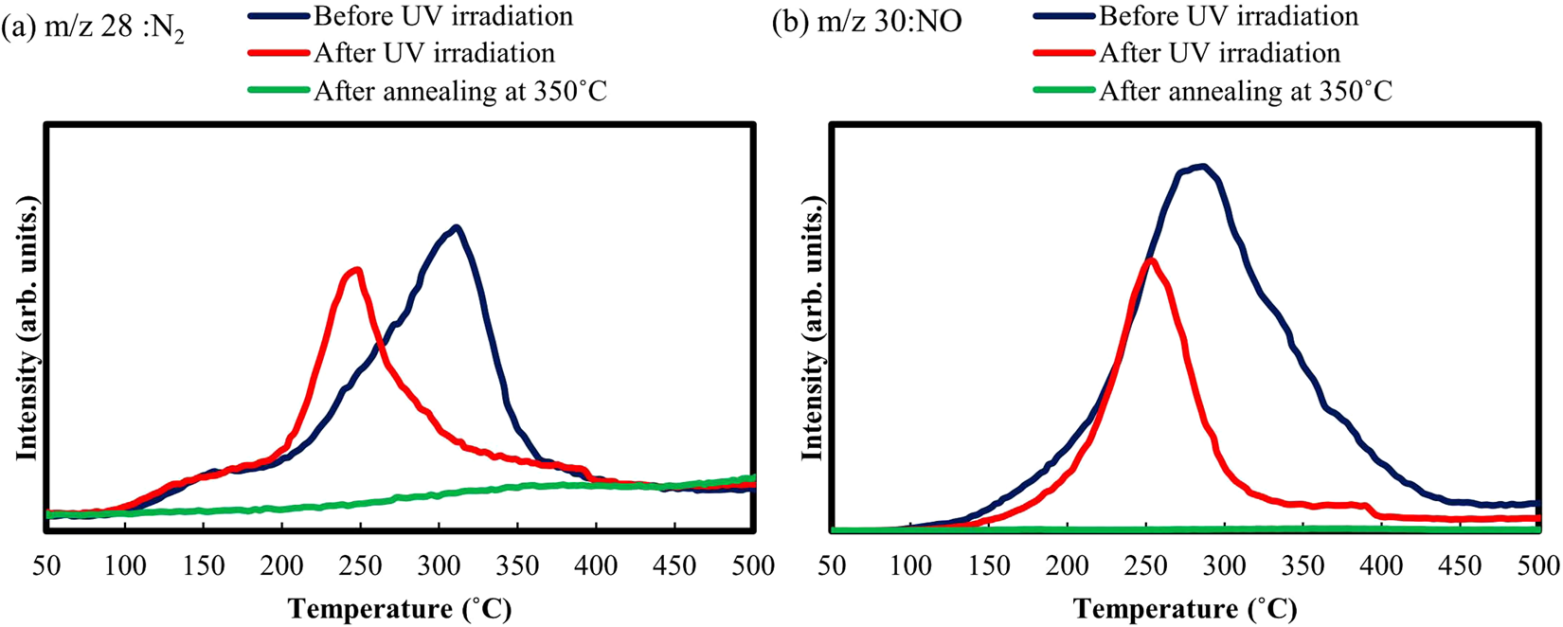
TDS spectra related to m/z of (**a**) 28 (N_2_) and (**b**) 30 (NO) for IGZO films by the direct-patterning process at the indicated conditions.

To analyze the photoreaction during direct patterning, fabricated IGZO films were evaluated by X-ray photoelectron spectroscopy [XPS; Fig. 4(a–d)]. Since the residual species were limited to nitrate ligands, changes in the N1s nitrate peaks were analyzed. O1s peaks were also analyzed by curve-fitting the M-O (lattice oxygen), Vo (oxygen vacancy), and M-OH (hydroxyl) peaks at 530.2, 531.7, and 533.0 eV, respectively^29^. As Fig. 4(a) shows, UV irradiation resulted in a decrease in nitrate peaks related to NO_3_, indicating that UV irradiation effectively decomposed the nitrate ligands. The XPS spectra also show that the M-O bonds were enhanced by UV irradiation. For confirmation, we conducted thermal desorption spectroscopy (TDS) analysis of the IGZO films. The desorption of nitrogen compounds was assessed by tracking the mass fragments at m/z values of 28 and 30, which corresponded with N2 and NO from the precursor compounds. Figure 5 shows the TDS spectra related to m/z of 28 (N2) and 30 (NO). A large amount of desorption signals that peaked at around 300 °C was observed in the IGZO film before UV irradiation. These desorption signals were related to the nitrate ligands because the soft-annealing temperature of 60 °C was very low. A considerable decrease in desorption was confirmed for the IGZO films after UV irradiation. UV irradiation was effective in decomposing the nitrate ligands and oxidizing the precursors. According to previous reports, UV irradiation effectively induced oxidation reaction and decomposition of ligands exclusively by photoreaction of the nitrate ligands^33^. In the proposed aqueous nitrate based precursor, the photochemical reactions based on water and nitrated ligands are induced to generate oxidizing agents. In the light of the XPS and TDS results, decomposition of nitrate ligands and enhanced M-O bonding formation contributed to changes in film solubility, which are important in the subsequent etching process.

**Figure 6:**
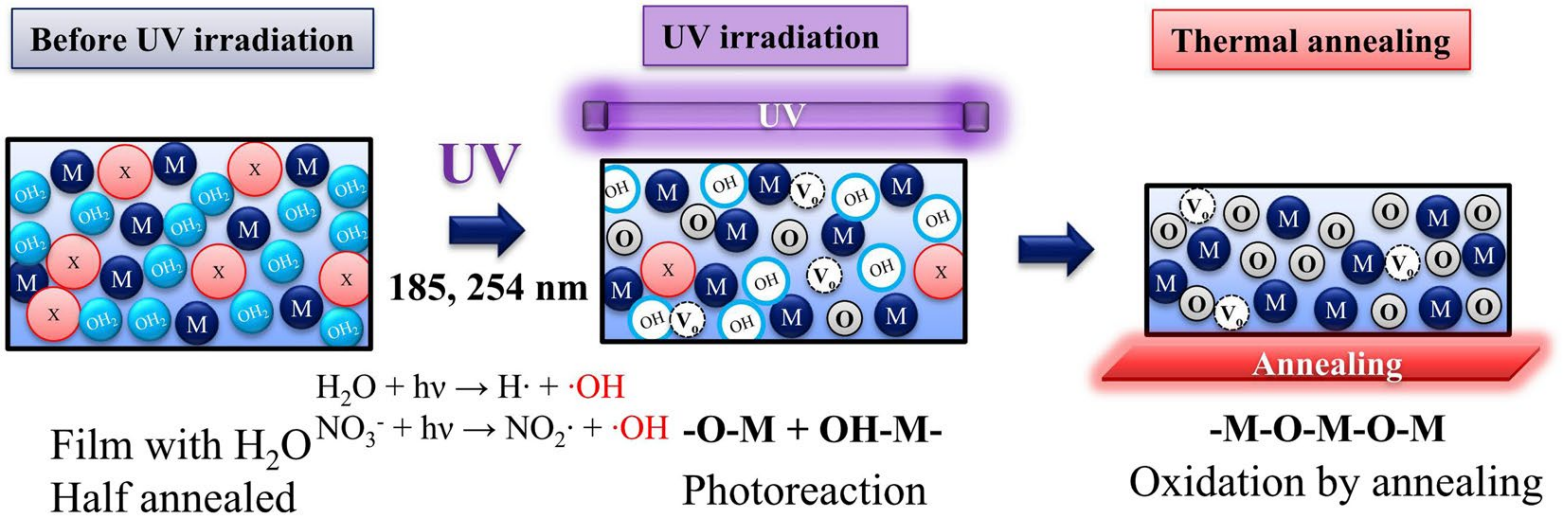
Schematic model of the oxidation reaction of metal complexes using the direct photoreactive patterning method.

Figure 6 shows a schematic model of the photoreaction based on the photodissociation of water molecules in the aqueous precursor followed by oxidation via thermal annealing. As indicated above, the photodissociation of water molecules and nitrate ligands under UV irradiation generated hydroxyl radicals, which acted as a strong oxidant for forming M-O bonds. Compared to the films annealed at 350 °C for 1 h, the formation of the M-O bonds after UV irradiation was insufficient in points of low intensity of the M-O bonds. However, the difference in partial oxidation between the irradiated and un-irradiated films was sufficient to achieve patterning using the etching process. Although the IGZO films after UV irradiation were not completely oxidized, the ensuing thermal annealing process enhanced the formation of metal-oxide-metal bonds and decomposed the impurities of metal ligands.

**Figure 7:**
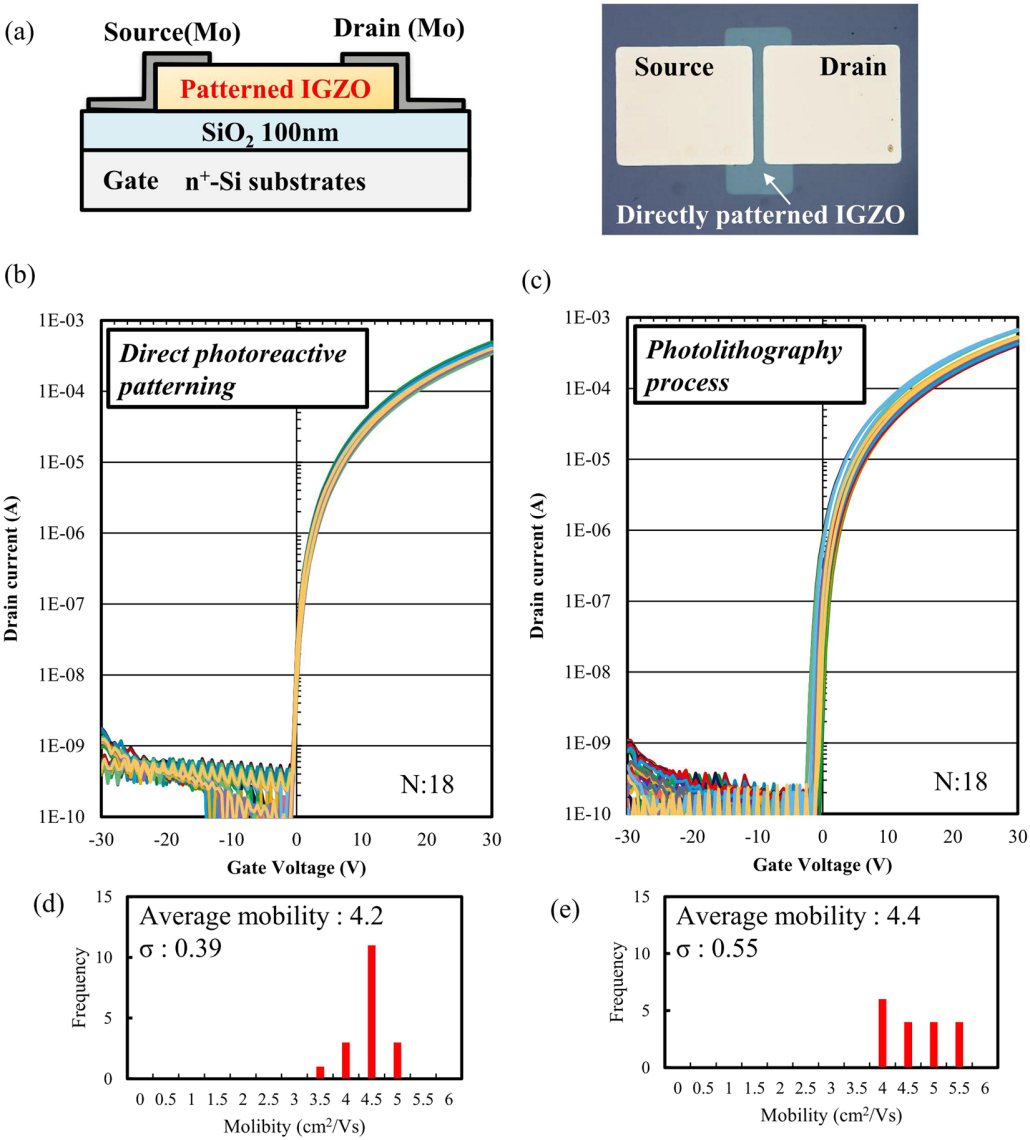
(**a**) Schematic diagram of a bottom-gate, top-contact TFTs and photographic image of a fabricated TFT. Transfer characteristics for 18 IGZO TFTs fabricated by (**b**) the direct photoreactive patterning process and (**c**) conventional photolithography. Mobility histograms of the TFTs fabricated by (**d**) direct patterning and (**e**) photolithography.

Figure 7(a–e) shows the transfer characteristics of the 18 IGZO TFTs fabricated by direct patterning and by conventional photolithography at gate voltages (*V*_g_) ranging from −30 to 30 V and a fixed drain voltage (*V*_d_) of 30 V. Table 1 summarizes the calculated electrical properties, including mobility (*μ*), ratio of maximum on-current to minimum off-current (*I*_on_/*I*_off_), subthreshold swing (S.S), switch-on voltage (*V*_on_) @ *I*_ds_ = 10^−9^ A, and hysteresis of *V*_on_. The calculated average mobility of the TFTs fabricated by direct patterning was 4.2 cm^2^/V·s with only a small deviation in mobility. Hysteresis in the switching behavior was also negligible. These results indicate robust uniform performance in conjunction with a high *I*_on_/*I*_off_ and minimal hysteresis. The ready decomposition and lack of impurities in the carbon-free aqueous precursor is believed to have contributed to the films‘ stable electrical characteristics. Direct patterning, compared to conventional photolithography, did not produce any film-degradation effects and yielded smaller deviations in the TFTs‘ electrical performance (based on mobility, *V*_on_, and hysteresis). The surface state for the back channel of oxide TFT is known as very sensitive and important layer to improve the performance of oxide TFTs^41,42^. To investigate the differences in the chemical states of the films fabricated by direct patterning vs. photolithography, FT-IR spectroscopy were conducted. As Fig. S3(b) shows, the increase in OH groups in the semiconductor layers of the films were examined and compared. As Fig. S3(c) shows, unique residual peaks indicating the presence of silazanes (Si-CH_3_), a residue comprising photoresist material as adhesion promoter^43^, were detected. One possible explanation for the larger deviation is attributed to the negative effect of the residual in the back channel during photolithography. Residues in the back channel can lead to undesirable back channel effects and require additional passivation processes^44^. In contrast, direct patterning does not require any chemicals which were consisted of various organic compounds. In addition, in the direct patterning process, high temperature thermal annealing (350 °C) of the IGZO films is performed after patterning, whereas in photolithography, annealing occurs before patterning. The difference of process flows is considered as another reason to obtain smaller deviations of TFT performance. Thus, the chemical state at the surface of TFT by direct patterning is considered to be more clean and stable.

**Table 1:** Calculated electrical parameters of solution-processed IGZO TFTs fabricated by the direct photoreactive patterning process and by conventional photolithography.

	Mobility (cm^2^/V·s)	*I*_on_/*I*_off_ ratio	S.S (V/decade)	*V*_on_ (V)	Hysteresis (V)
Direct photoreactive patterning	4.2	−10^6^	0.4	−0.4	0.1
	σ: 0.4		σ: 0.03	σ: 0.07	σ: 0.04
Conventional photolithography	4.4	−10^6^	0.4	−1.3	0.7
	σ: 0.6		σ: 0.04	σ: 0.48	σ: 0.20

**Figure 8:**
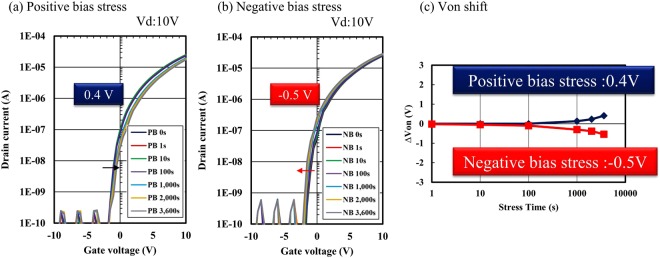
Transfer characteristics of a direct-patterned IGZO TFT under (**a**) positive bias stress and (**b**) negative bias stress at an applied drain voltage of 10 V. (**c**) Changes in switch-on voltage as a function of cumulative stress time for positive and negative bias stresses.

Finally, to evaluate theelectrical stability of the IGZO TFTs fabricated by direct patterning, both positive gate bias stress (PBS) and negative bias stress (NBS) were assessed. Figure 8 shows the transfer characteristics of the direct-patterned IGZO TFTs under PBS and NBS. To suppress the interaction between the air atmosphere and back channels of the TFTs, the back channels were passivated with a coating-type insulation layer, and the transfer characteristics of the TFTs at *V*_g_ ranging from −15 to 15 V were assessed at an applied drain voltage of 10 V. The stress conditions for PBS and NBS were ± 1 MV/cm, and the maximum stress time was 3,600 s at room temperature. The direct-patterned TFTs exhibited reliable switch-on voltage with Δ*V*_on_ less than 1.0 V under both PBS and NBS. It is found that the aqueous solution-processed TFTs fabricated via simple direct photoreactive patterning method exhibit stable reliability. It is likely that the simple carbon-free aqueous precursor with the simple patterning process, which involves few steps without organic solvents, achieved good stability under bias stress condition.

**Figure 9:**
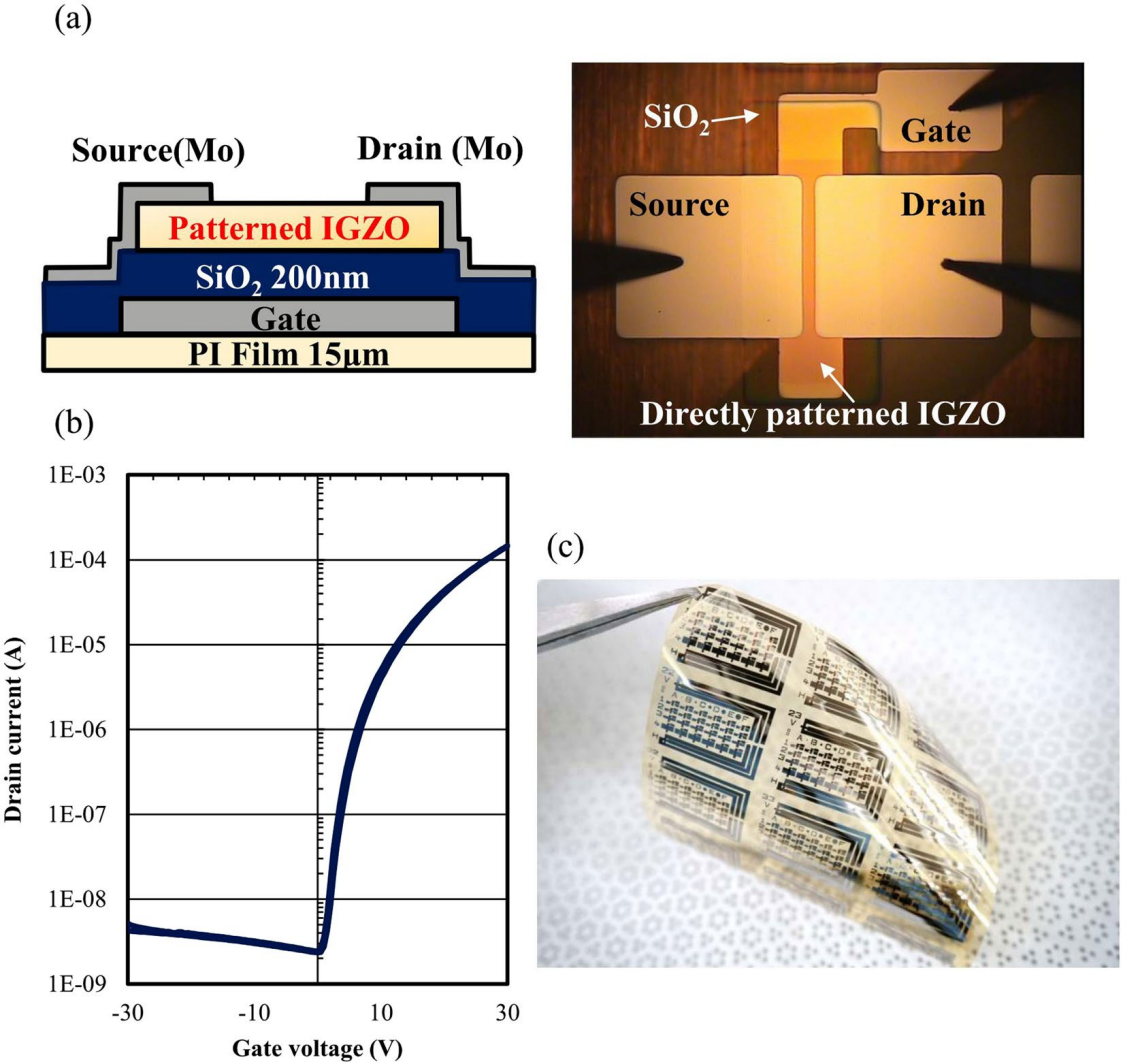
(**a**) Schematic diagram of a bottom-gate, top-contact TFTs and photographic image of a fabricated TFT on flexible polyimide substrate. (**b**) Transfer characteristics for IGZO TFT fabricated by the direct photoreactive patterning process. (**c**) Photograph of IGZO TFTs on the flexible polyimide substrate.

To confirm the compatibility of direct patterning for flexible electronics, we fabricated a flexible IGZO TFTs using the direct patterning method. As Fig. 9 shows, a bottom-gate, top-contact IGZO TFT was fabricated on top a flexible polyimide substrate. The calculated mobility of the TFT was 4.8 cm^2^/V·s. We confirmed that the proposed direct patterning method was suitable for flexible electronics.

## Conclusion

We used a simple and easy photoreactive direct-patterning method to develop a simple and reliable direct patterning method for carbon-free aqueous solution-processed IGZO TFTs. The patterning method is achieved by the photooxidation of water molecules and nitrate ligands. XPS and TDS analysis indicated that UV irradiation leads to the dissociation of nitrate ligands and oxide formation, altering the film solubility for the subsequent etching process. The fabricated TFTs annealed at 350 °C exhibited good electrical performance and uniformity. The mobilities of the fabricated TFTs were uniformed, with an average value of 4.3 cm^2^/V·s and small deviations. Since the proposed photoreactive patterning method is simple in its mechanism, namely the photooxidation of water molecules and nitrate ligands, we expect the method to be applicable to various types of aqueous metal oxide precursors, including semiconductors, dielectric oxide materials, and other conductive materials. In addition, given the environmental benefits of this method, we expect the method to be used in various electrical device applications.

## Methods

### Precursor synthesis

A 0.3 M IGZO precursor solution was prepared by dissolving indium nitrate hydrate [In(NO_3_)_3_·*x*H_2_O], gallium nitrate hydrate [Ga(NO_3_)_3_·*x*H_2_O], and zinc nitrate hydrate [Zn(NO_3_)_2_·*x*H_2_O] in pure water (Millipore system, resistivity: ~18 MΩ1 cm, 25 °C). The molar concentration of 0.3 M was achieved by setting the molar ratio of In:Ga:Zn to 4:1:1. Prior to the spin-coating process, the precursor was stirred vigorously to completely dissolve the precursors in solution. All the reagents were obtained from Aldrich and were used as-received without further purification.

### TFT fabrication

Solution-processed IGZO thin films were fabricated by spin-coating onto 100 nm thick, thermally oxidized SiO_2_/n^+^-Si substrates. The n^+^-Si substrate was used as the gate electrode, while the SiO_2_ layer was used as the dielectric. The substrate was treated with oxygen plasma for 30 s before spin-coating to enhance hydrophilicity. The precursor solution was passed through a 0.22 μm polytetrafluoroethylene filter. Spin-coating was performed at 4000 rpm for 30 s. Then, soft annealing was performed to retain H_2_O inside the films at 60 °C for 10 s. Then, the photoreaction was carried out using deep-UV irradiation system (UV-300, Samco Inc.) with the primary emission line of UV of 185 nm (10%) and 254 nm (90%) provided by a low-pressure mercury lamp for 10 min. The intensity of UV lamp was 0.8 mW/cm^2^ (250 nm detector, Orc Manufacturing Co., Ltd. with 2 cm of sample-to-lamp distance. The substrate temperature after 10 min UV irradiation was about 38 °C. N_2_ atmosphere was set to prevent the generation of ozone gas. Fine metal masks were used to pattern the desired island shapes. Etching was performed using dilute citric acid (1 wt%) for 30 s. After patterning, the films were annealed at 350 °C for 1 h in air. The thickness of the resulting film was approximately 10 nm. Finally, patterned molybdenum source/drain electrodes (50 nm) were formed using a shadow mask to obtain bottom-gate, top-contact IGZO TFTs with 1000 μm channel widths and 100 μm channel lengths. To evaluate for bias stability, the TFTs were encapsulated with a coating-type insulation layer (ZEOCOAT, cycloolefin resin, Zeon Corporation) to prevent interaction with the atmosphere at the back channel of the semiconductor layer.

TFTs by conventional photolithography process were also fabricated. Spin-coating was performed at 4000 rpm for 30 s, followed by pre-annealing at 150 °C for 10 min and subsequent annealing at 350 °C for 1 h in air. After fabrication of IGZO films, photolithography was performed using dilute hydrochloric acid (0.01 wt%) for wet etching. After removing the photoresist, the drying process was carried out at 150 °C for 30 min.

The IGZO TFTs on a flexible polyimide substrate were fabricated using polyimide varnish. A polyimide film was spin-coated on a glass substrate and a barrier layer was formed. After formation of the gate electrode of molybdenum (50 nm), a 200-nm-thick SiO_2_ gate insulator film was deposited by sputtering deposition. Then, the substrate was treated with oxygen plasma for 30 s to enhance hydrophilicity. Spin-coating of the IGZO film was performed. The direct-patterning process was performed by the same aforementioned method. After patterning, the films were annealed at 350 °C for 1 h in air. Then, the source and drain (S/D) electrodes were formed. Shadow masks were used to pattern all the layers of the TFTs. After fabrication of the TFTs, the polyimide film was mechanically peeled from the glass substrate.

### Characterization

The TFTs were evaluated in the dark and at room temperature using an electrical measurement system with a probe station and a semiconductor analyzer (Keithley HP4156C). The mobility is calculated as

$$Ids=\frac{W}{2L}{C}_{i}\mu {({V}_{g}-{V}_{t})}^{2},$$

where *C*_*i*_ is the capacitance of the gate dielectric per unit area, *W* is the channel width, and *L* is the channel length. The fabricated IGZO films were characterized by XPS (ULVAC-PHI PHI Quantera II), TDS (TDS; ESCO EMDWA1000S), and Fourier transform infrared spectroscopy (FT-IR; Bruker VERTEX 70 v). Film thicknesses were analyzed with a surface profilometer (Bruker, Dektak stylus profiler system). The etching rate was calculated from the etched depths after removal of the photoresist mask from the film.

## Electronic supplementary material

Supplementary Information
