# Supplementary material for: Simple and reliable direct patterning method for carbon-free solution-processed metal oxide TFTs
Source: Sci Rep. 2018 Aug 27;8:12825. doi: 10.1038/s41598-018-31134-w (PMC6110810; doi:10.1038/s41598-018-31134-w)
Supplement: Supplementary file 1 — Supplementary Information [file 41598_2018_31134_MOESM1_ESM.pdf]

Supplementary Information

Simple and reliable direct patterning method for carbon-free solution-processed metal oxide TFTs

Masashi Miyakawa*, Mitsuru Nakata, Hiroshi Tsuji, and Yoshihide Fujisaki
NHK Science & Technology Research Laboratories, Tokyo, 157-8510, Japan
*E-mail: miyakawa.m-eo@nhk.or.jp

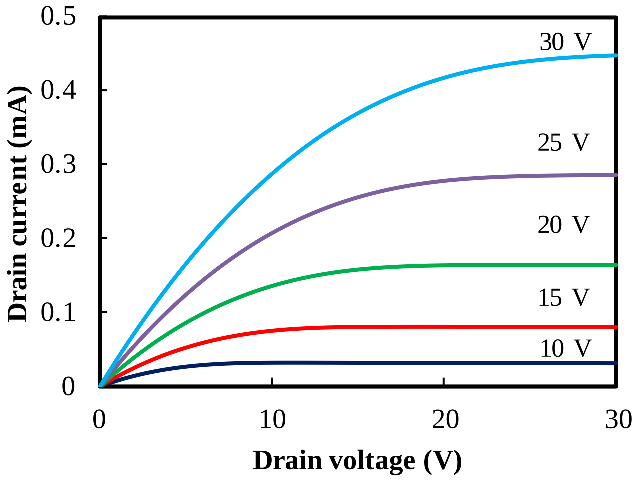

Figure S1. Output characteristics for a solution-processed IGZO TFT onto thermally oxidized SiO_2/n^+-Si substrates fabricated by the direct photoreactive patterning method.

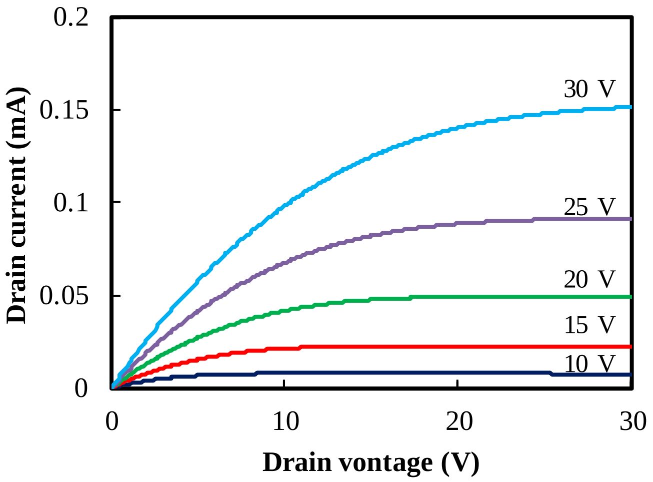

Figure S2. Output characteristics for a solution-processed IGZO TFT onto flexible substrate fabricated by the direct photoreactive patterning method.

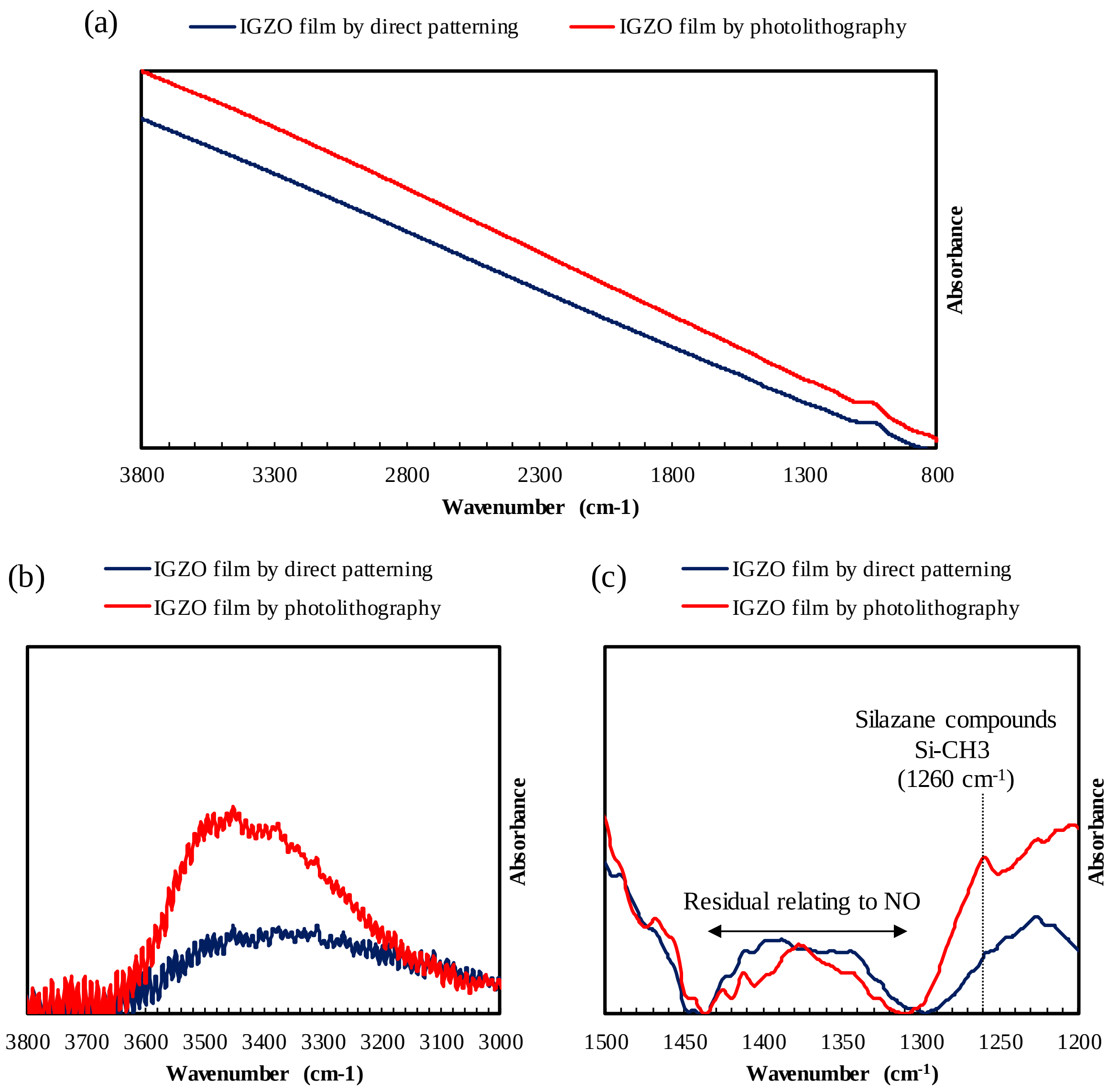

Figure S3. FT-IR spectra for the IGZO films by the direct photoreactive patterning process and conventional photolithography.
